# A previously unrecognized role of C3a in proteinuric progressive nephropathy

**DOI:** 10.1038/srep28445

**Published:** 2016-06-27

**Authors:** Marina Morigi, Monica Locatelli, Cinzia Rota, Simona Buelli, Daniela Corna, Paola Rizzo, Mauro Abbate, Debora Conti, Luca Perico, Lorena Longaretti, Ariela Benigni, Carlamaria Zoja, Giuseppe Remuzzi

**Affiliations:** 1 IRCCS - Istituto di Ricerche Farmacologiche “Mario Negri”, Centro Anna Maria Astori, Science and Technology Park Kilometro Rosso, Bergamo, Italy; 2 Unit of Nephrology and Dialysis, Azienda Socio Sanitaria Territoriale (ASST) Papa Giovanni XXIII, Bergamo, Italy; 3 Department of Biomedical and Clinical Sciences, University of Milan, Milan, Italy

## Abstract

Podocyte loss is the initial event in the development of glomerulosclerosis, the structural hallmark of progressive proteinuric nephropathies. Understanding mechanisms underlying glomerular injury is the key challenge for identifying novel therapeutic targets. In mice with protein-overload induced by bovine serum albumin (BSA), we evaluated whether the alternative pathway (AP) of complement mediated podocyte depletion and podocyte-dependent parietal epithelial cell (PEC) activation causing glomerulosclerosis. Factor H (C*fh*^−/−^) or factor B-deficient mice were studied in comparison with wild-type (WT) littermates. WT+BSA mice showed podocyte depletion accompanied by glomerular complement C3 and C3a deposits, PEC migration to capillary tuft, proliferation, and glomerulosclerosis. These changes were more prominent in C*fh*^−/−^ +BSA mice. The pathogenic role of AP was documented by data that factor B deficiency preserved glomerular integrity. In protein-overload mice, PEC dysregulation was associated with upregulation of CXCR4 and GDNF/c-Ret axis. *In vitro* studies provided additional evidence of a direct action of C3a on proliferation and CXCR4-related migration of PECs. These effects were enhanced by podocyte-derived GDNF. In patients with proteinuric nephropathy, glomerular C3/C3a paralleled PEC activation, CXCR4 and GDNF upregulation. These results indicate that mechanistically uncontrolled AP complement activation is not dispensable for podocyte-dependent PEC activation resulting in glomerulosclerosis.

The progression of proteinuric nephropathies to end stage renal failure represents a major health problem worldwide. Glomerulosclerosis -i.e. extracellular matrix accumulation and fibrosis that progressively occupy the glomerular tuft- and tubulointerstitial damage, are the structural hallmarks of the process^1^,^2^,^3^. Clarifying the mechanisms underlying glomerular structural and functional damage in progressive nephropathies might help to identify potential novel therapeutic strategies.

The general consensus, derived from experimental^4^,^5^,^6^,^7^,^8^,^9^,^10^ and human studies^11^,^12^,^13^,^14^,^15^, recognizes that podocyte injury and loss is the initial event in a complex process that ends in glomerulosclerosis. Podocytes are postmitotic cells with limited capacity to proliferate and are considered almost incapable of replenishing their numbers following loss in disease^16^,^17^. Recent evidence, however, has challenged this paradigm, indicating that the neighboring glomerular parietal epithelial cells (PECs) might represent a reserve of podocyte progenitors^18^,^19^,^20^,^21^,^22^. Thus, PEC differentiation towards podocytes has been described during kidney development, in the early postnatal life of rodents and humans^23^,^24^, conceivably to compensate for possible turnover after a mild injury. Studies in animal models of progressive glomerulopathy^25^,^26^ and genetic-cell tracking experiments^6^,^7^ document that podocyte loss exceeding a certain threshold leads to the appearance of denuded areas of glomerular basement membrane (GBM), resulting in adhesion of the glomerular capillary loop to Bowman’s capsule^8^. This induces aberrant behaviour in PECs^27^, which start to proliferate and produce extracellular matrix, driving the formation of hyperplastic and sclerotic lesions^25^,^27^,^28^,^29^,^30^,^31^. Among the factors that influence the maladaptive PEC response, ultrafiltered albumin, an individual component of proteinuria, has been regarded as a critical player that impairs podocyte regeneration^32^. Notably, albumin prevents PEC differentiation into podocytes by sequestering retinoic acid and impairing retinoic acid response element (RARE)-mediated transcription of podocyte specific genes^32^. Circulating components of the complement system are also lost in the urine in proteinuric conditions and become activated at the glomerular level, favoring progression of the lesions^33^,^34^,^35^,^36^. Thus, abnormal fixation of ultrafiltered C3 is detected in podocytes showing signs of dedifferentiation and injury during the early stage of proteinuric disease in rats with remnant kidney^33^, and in mice with protein-overload proteinuria^36^. C3 deficient mice with protein-overload are protected against podocyte structural damage and sclerosis, indicating that C3 might increase susceptibility to injury. Moreover, results from transplantation studies revealed that circulating but not renal synthesized C3 contributes to the development of glomerular sclerotic lesions. A milder disease is induced by protein-overload in wild-type kidneys grafted into C3-deficient mice compared to C3^−/−^ kidneys given to complement-competent mice^36^.

However, a number of questions remain: 1. Does ultrafiltered complement favor podocyte depletion in the presence of proteinuria in such a way that PECs become activated? 2. Which pathway of complement activation is involved? 3. Which, if any, are the podocyte-derived proteins triggered by complement activation responsible for disease progression? Answering these questions is of particular clinical relevance given the current availability of complement inhibitor therapies, tested in patients with several complement-related diseases, such as paroxysmal nocturnal hemoglobinuria, atypical hemolytic uremic syndrome and age-related macular degeneration, which have spectacular benefit^37^,^38^,^39^,^40^,^41^.

Here we demonstrate that complement activation via alternative pathway (AP) is a pivotal trigger for podocyte loss and PEC activation, leading to glomerulosclerosis in the model of protein-overload proteinuria. This is a particularly suitable model for this purpose in that it is not immunologically mediated as documented convincingly by previous studies^36^,^42^. *In vivo* experiments were based on mice deficient in factor H, a complement regulator that inhibits AP activation, and mice deficient in factor B, an essential component of the AP-related C3 and C5 convertases^43^. *In vitro* data underlined the importance of C3a as a potent activator of PEC migration and proliferation, an effect further potentiated by complement-activated podocytes releasing GDNF. The aberrant regulation of PECs by complement and the GDNF/c-Ret axis highlights the importance of these pathways in altering phenotype of glomerular PECs and opens unanticipated therapeutic perspectives.

## Results

### *In vivo* studies

#### Complement activation via the alternative pathway induces podocyte loss

**Figure 1:**
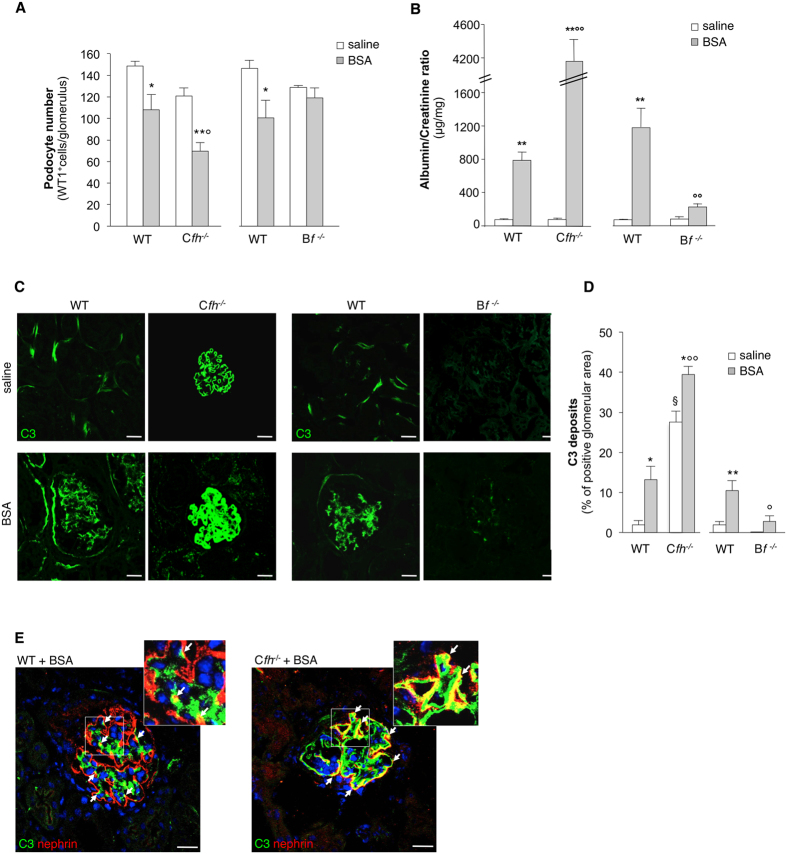
Complement activation via AP causes podocyte depletion in mice with protein-overload proteinuria. (**A**) Quantification of WT1-positive podocytes expressed as number per glomerulus in C*fh*^−/−^, B*f*^−/−^ mice and the corresponding WT littermates receiving saline or BSA (n = 5 mice/group), evaluated on day 23 after starting BSA. *P < 0.05, **P < 0.01 versus corresponding saline; ^°^P < 0.05 vs corresponding WT+BSA. (**B**) Urinary albumin/creatinine ratio measured on day 23 after starting BSA in WT, C*fh*^−/−^ and B*f*^−/− ^mice receiving saline or BSA. **P < 0.01 versus corresponding saline; °°P < 0.01 vs corresponding WT+BSA (*n* = 4 WT mice treated with saline or BSA, *n* = 5 C*fh*^−/−^ +saline mice, *n* = 6 C*fh*^−/−^ +BSA mice and *n* = 5 B*f*^−/−^ mice treated with saline or BSA). (**C**) Representative images showing glomerular C3 deposits (green) in WT, C*fh*^−/−^ or B*f*^−/−^ mice injected with saline or BSA. Scale bars: 20 μm. (**D**) Quantification of C3 staining expressed as percentage of positive glomerular area. *P < 0.05, **P < 0.01 versus corresponding saline; ^§^P < 0.0001 versus corresponding WT+saline, °P < 0.01, °°P < 0.0001 versus corresponding WT+BSA (*n* = 4 WT mice treated with saline or BSA, *n* = 5 C*fh*^−/−^ mice treated with saline or BSA and *n* = 5 B*f*^−/−^ mice treated with saline or BSA). (**E**) Representative images of renal tissue from WT and C*fh*^−/−^ +BSA mice showing merged area (yellow) of costaining of glomerular C3 deposits (green) and nephrin (red) in podocytes. Insets display enlarged images. Nuclei are counterstained with DAPI. Scale bars: 20 μm. Data information: Values are presented as mean ± SEM. ANOVA corrected with Tukey (**B**) or Bonferroni (**A**,**C**,**D**,**E**) coefficient.

The role of complement was studied in the model of protein-overload proteinuria developed in mice deficient in factor H (C*fh*^−/−^)^44^ and factor B (B*f*^−/−^)^45^, and the corresponding wild-type (WT) littermates. In both groups of WT mice injected with BSA, a significant reduction in the number of podocytes, labeled for the specific marker WT1, was detected compared to saline (28% and 31% decrease, P < 0.05) (Fig. 1A). This result was confirmed by staining for nestin, another podocyte marker^26^,^46^ (nestin expression: WT+BSA littermates of C*fh*^−/−^, 4.52 ± 0.67 versus saline, 6.89 ± 0.45%, P < 0.05; WT+BSA littermates of B*f*^−/−^, 3.38 ± 0.25 versus saline, 5.33 ± 0.51% glomerular area, P < 0.05) (Figure S1A). Abnormal passage of proteins across the glomerular filtering barrier in both groups of WT+BSA mice (Fig. 1B) resulted in C3 deposition within glomeruli, which was barely absent in mice injected with saline (Fig. 1C,D). C3 was found in the glomerular capillary walls and accumulated in the context of podocytes, as evidenced by nephrin staining (Fig. 1E). C*fh*^−/−^ +BSA mice showed a significant reduction (42%) in podocyte number (Fig. 1A, P < 0.01 versus saline) associated with marked increase in urinary albumin/creatinine ratio compared to the corresponding WT+BSA mice (Fig. 1B). In C*fh*^−/−^ mice, linear C3 deposits were present within the glomerular capillaries (Fig. 1C). After BSA, C3 accumulation increased (Fig. 1C,D) at podocyte level, as shown by C3 costaining with nephrin (Fig. 1E). By electron microscopy analysis, subendothelial dense deposits are typical features in C*fh*^−/−^ mice (Figure S1B). The ultrastructural analysis of podocytes of C*fh*^−/−^ +BSA mice showed protein droplets, vacuoles, prominent cytoskeleton rearrangement associated with foot process effacement, and microvillous transformation, absent in C*fh*^−/−^ mice given saline (Figure S1B).

To establish a mechanistic role of complement activation via AP on podocyte loss, we took advantage of mice deficient in factor B^45^. C3 staining was weak or absent in the kidneys of B*f*^−/−^ mice given BSA (Fig. 1C,D). Notably, in this setting podocyte number was preserved (Fig. 1A), in association with lower urinary protein excretion compared with WT+BSA mice (Fig. 1B). There was no or trace glomerular staining for C4 in all the experimental groups (Figure S2), thus excluding the involvement of the classical pathway of complement activation in the protein-overload model.

#### Complement activation mediates parietal epithelial cell dysregulation

**Figure 2:**
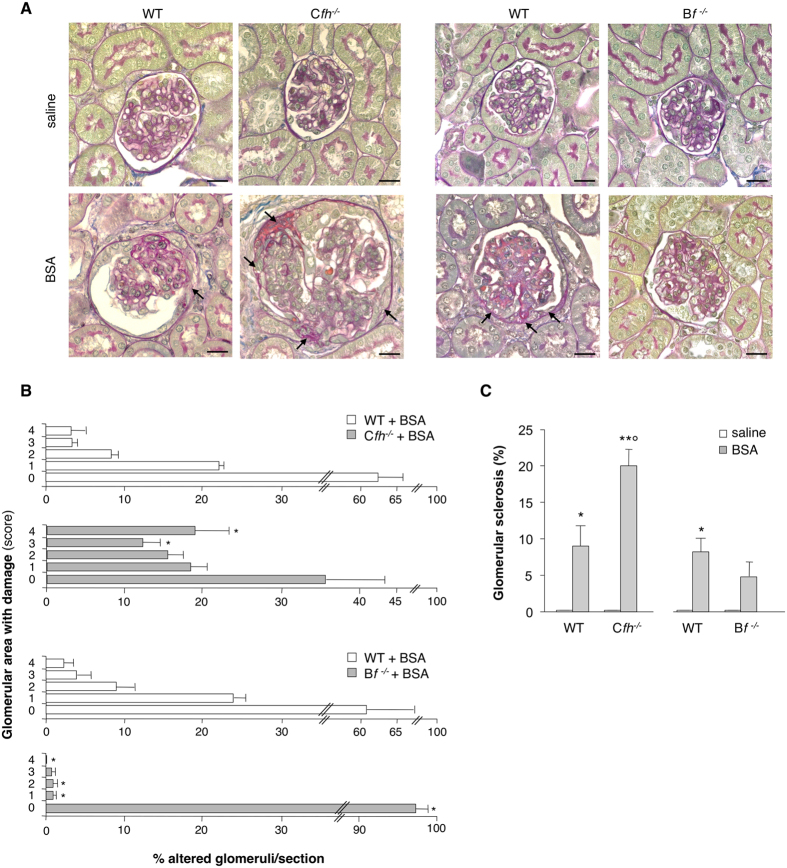
Uncontrolled complement deposition fosters glomerular hyperplastic-like lesions in protein-overload proteinuria. (**A**) PAS-stained images of representative glomeruli from C*fh*^−/−^, B*f*^−/−^ mice and the corresponding WT littermates receiving saline or BSA. Arrows indicate area of adhesion between the glomerular tuft and Bowman’s capsule. Scale bars: 20 μm. (**B**) Quantification of percentage of glomeruli with different degrees of glomerular lesions/adhesions, scored from 0 to 4, where 0 is without lesions, 1 ≤ 25%, 2 = 26–50%, 3 = 51–75%, 4 ≥ 76% of area of adhesion between the glomerular tuft and Bowman’s capsule. *P < 0.05 versus corresponding WT+BSA by non-parametric Mann-Whitney test (*n* = 4 corresponding WT littermates, *n* = 6 C*fh*^−/−^ and *n* = 5 B*f*^−/−^ mice/group treated with BSA). (**C**) Percentage of glomeruli affected by sclerotic lesions assessed in PAS-stained kidney sections of WT, C*fh*^−/−^ and B*f*^−/−^ mice treated with saline or BSA. *P < 0.05, **P < 0.01 versus corresponding saline, °P < 0.01 versus corresponding WT+BSA by ANOVA corrected with Bonferroni coefficient (*n* = 4 WT+saline mice, n = 5 WT+BSA mice, *n* = 5 C*fh*^−/−^ +saline mice, *n* = 6 C*fh*^−/−^ +BSA mice and *n* = 5 B*f*^−/−^ mice treated with saline or BSA). Data information: Values are presented as mean ± SEM.

In several glomerular diseases podocyte dysfunction is associated with dysregulation of PECs, a condition which favors the generation of synechiae, early adhesions between the glomerular tuft and the Bowman’s capsule that evolve to sclerosis^25^,^26^,^27^,^29^. Examination of PAS-stained kidney sections showed normal glomerular structure (scored 0) in any group of mice given saline (Fig. 2A). In WT littermates of C*fh*^−/−^ or B*f*^−/−^ mice that received BSA, the percentage of total glomeruli that showed adhesions (scored 1 to 4) between the glomerular tuft and the Bowman’s capsule averaged 38 ± 4% and 39 ± 8%, respectively (Fig. 2A,B). Exaggerated complement activation in C*fh*^−/−^ +BSA mice was associated with more widespread glomerular adhesions (65 ± 9% of glomeruli), and more sclerotic lesions compared to the corresponding WT+BSA mice (Fig. 2). At variance, in B*f*^−/−^ mice only mild glomerular lesions were observed after BSA (Fig. 2).

**Figure 3:**
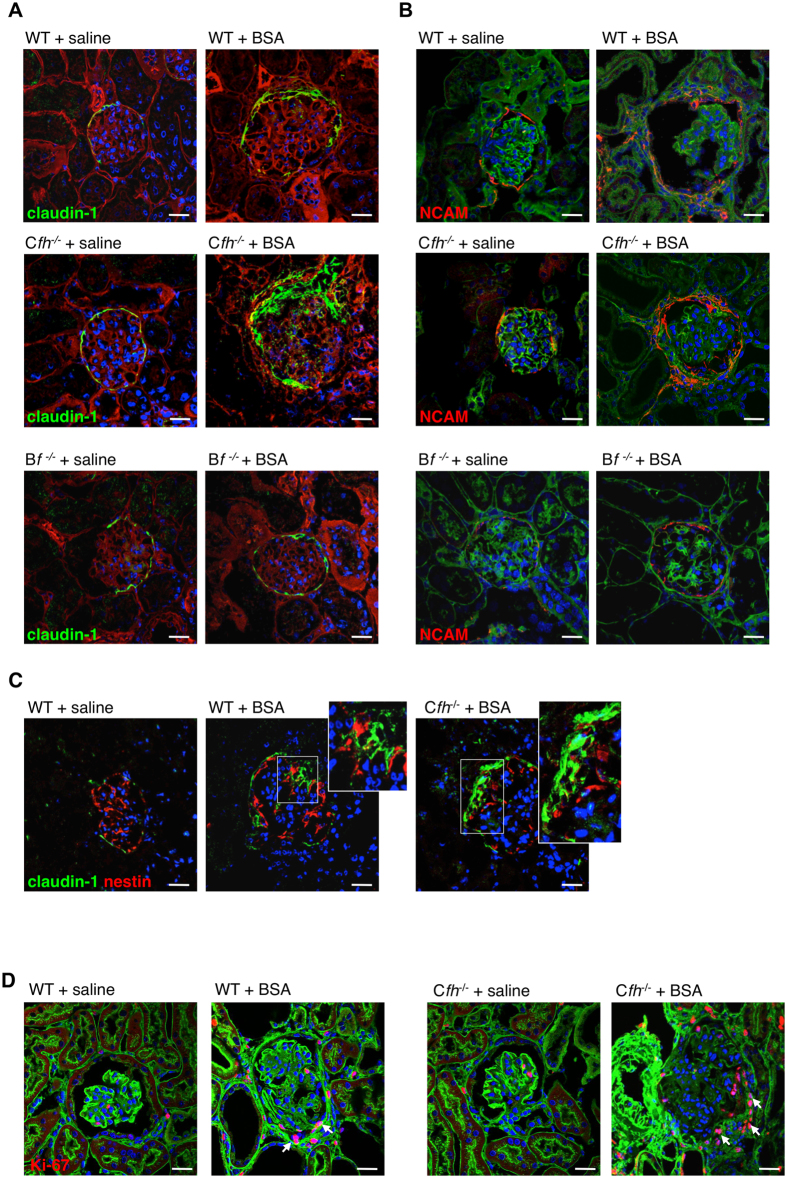
Complement activation induces PEC dysregulation and proliferation in response to protein-overload. (**A,B**) Representative images showing claudin-1 (A, green) and NCAM (B, red) expression in WT (littermates of C*fh*^−/−^ mice), C*fh*^−/−^ and B*f*^−/−^ mice injected with saline or BSA. Renal structure is stained with rhodamine (**A**, red) or FITC-WGA lectin (**B**, green). (**C**) Double immunofluorescence staining for claudin-1 (green) and nestin (red) in WT mice given saline and BSA and in C*fh*^−/−^ mice treated with BSA. Insets display enlarged images of claudin-1^+^ PECs and nestin^+^ podocytes in the context of glomerular lesions. (**D**) Representative immunofluorescence images showing Ki-67-positive cells (red, arrows) in WT and C*fh*^−/−^ mice injected with saline or BSA. Renal structure is counterstained with FITC-WGA lectin (green). Data information: Nuclei are counterstained with DAPI (blue). Scale bars: 20 μm.

To define the relative contribution of PECs and podocytes to the progression of complement-dependent glomerular lesions, we evaluated the expression of claudin-1, an intercellular tight junction protein constitutively expressed by all PECs lining the Bowman’s capsule^47^, and neural cell adhesion molecule (NCAM), a marker expressed exclusively by PECs with progenitor cell phenotype in rodents^25^,^26^,^48^. After protein-overload, WT littermates of C*fh*^−/−^ mice exhibited altered distribution of claudin-1-positive PECs that localized in areas of adhesions of glomerular capillaries to the Bowman’s capsule (Fig. 3A). NCAM-positive cells contributed substantially to glomerular hyperplastic-like lesions (Fig. 3B). In C*fh*^−/−^ +BSA mice a more severe PEC activation, consisting of multiple layers of claudin-1-positive and NCAM-positive PECs at the sites of glomerular lesions, was observed (Fig. 3A,B). Analysis of nestin-positive cells revealed that podocytes participated to a lesser extent than claudin-1-positive PECs to the above described glomerular lesions both in WT and C*fh*^−/−^ +BSA mice (Fig. 3C). At variance, no changes in the distribution of the two glomerular cell populations were observed in B*f*^−/−^ +BSA mice (Fig. 3A,B; Figure S3A–C), indicating that complement cascade activation via the AP is the key step towards glomerular structural alterations.

#### Complement activation promotes PEC proliferation and glomerular upregulation of CXCR4 and GDNF

**Figure 4:**
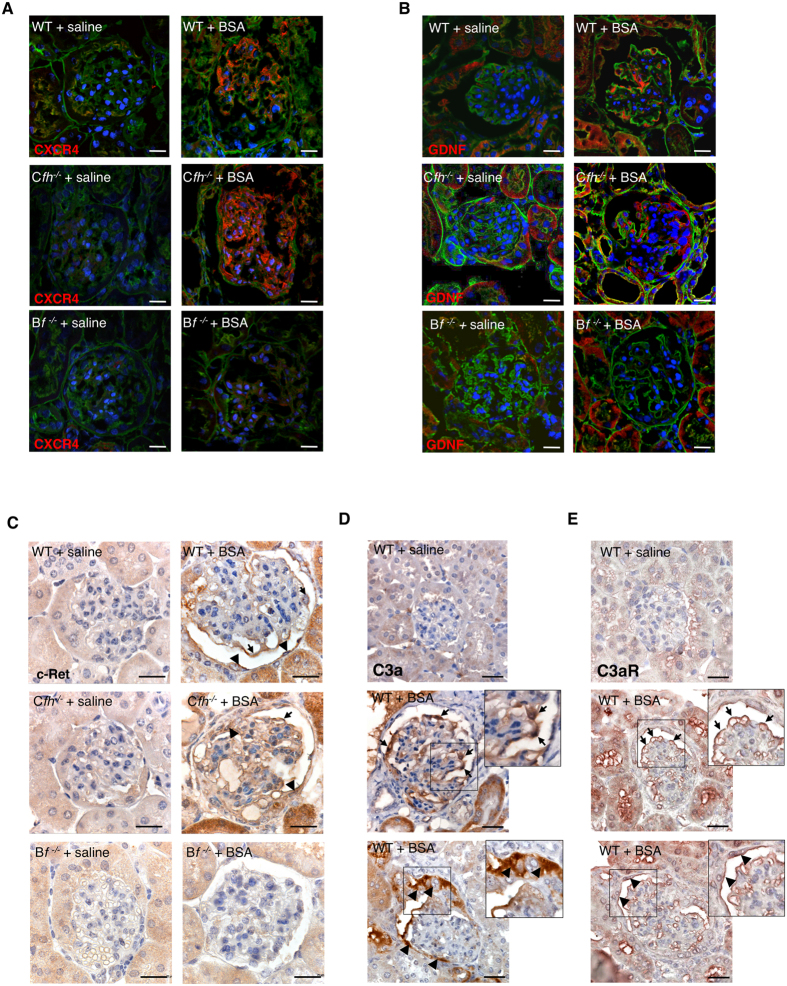
Complement activation, paralleled by glomerular C3a/C3a receptor expression, induces the expression of CXCR4 and GDNF in protein-overloaded mice. (**A,B**) Expression of CXCR4 (**A**, red) and GDNF (**B**, red) in kidney samples of WT (littermates of C*fh*^−/−^ mice), C*fh*^−/−^ and B*f*^−/−^ mice injected with saline or BSA. Renal structure is counterstained with FITC-WGA lectin (green). (**C**) Glomerular expression of c-Ret in podocytes (arrows) and PECs (arrowheads) by immunohistochemistry in WT (littermates of C*fh*^−/−^ mice), C*fh*^−/−^ and B*f*^−/−^ mice injected with saline or BSA. (**D,E**) Representative images of C3a (**D**) and C3a receptor (**E**) by immunohistochemistry in WT mice injected with saline or BSA. Podocytes and PECs are indicated with arrows and arrowheads, respectively. Insets display enlarged images. Data information: Nuclei are counterstained with DAPI (blue). Scale bars: 20 μm.

The role of complement on PEC proliferation was defined by studying Ki-67 expression. Very few PECs positive for Ki-67 were present in the Bowman’s capsule of WT mice given saline (Fig. 3D). After BSA, an increased number of proliferating cells was detected along the Bowman’s capsule at the site of glomerular lesions in WT and even more in C*fh*^−/−^ mice (Fig. 3D). Moreover, upregulation of CXCR4, a receptor involved in cell migration^31^,^49^ was observed in both podocytes and PECs in WT+BSA mice and, to a greater extent, in C*fh*^−/−^ +BSA mice (Fig. 4A). We then evaluated the glomerular expression of GDNF, a factor with mitogenic and promigratory properties^50^,^51^. A marked increase in GDNF expression was found in the podocytes of WT+BSA mice and to a higher degree in C*fh*^−/−^ +BSA mice, which also revealed GDNF positivity in PECs (Fig. 4B). In parallel, the expression of its receptor c-Ret was enhanced in podocytes and PECs of mice with protein-overload (Fig. 4C). These data possibly reflect a shift of PECs towards a proliferative and pro-migratory phenotype caused by an exaggerated glomerular complement activation, as supported by the evidence that the blockade of complement activation in B*f*^−/−^ +BSA mice prevented cell proliferation (Figure S3D) and maintained glomerular espression of CXCR4, GDNF and c-Ret at control levels (Fig. 4A–C; Figure S3E–G).

#### Glomerular expression of C3a and C3a receptor in mice with protein-overload proteinuria

Since complement activation via AP and cleavage of C3 represent a critical step in the generation of the active fragment C3a^43^, we investigated the expression of C3a and its receptor, C3aR, in glomeruli of mice with protein-overload proteinuria. As shown in Fig. 4D, no signal for C3a was detected in WT mice given saline. After BSA, positive staining for C3a was observed in podocytes (Fig. 4D, arrows) and in PECs along the Bowman’s capsule as well as in the hyperplastic-like lesions (Fig. 4D, arrowheads). The presence of C3a was coupled with increased expression of C3aR by podocytes and PECs (Fig. 4E, arrows and arrowheads, respectively) in glomeruli of WT+BSA mice. Consistently, in C*fh*^−/−^ mice with BSA the expression of C3a and C3aR was enhanced both in podocytes and PECs compared with corresponding animals given saline (Figure S4A,B). At variance, no glomerular staining for C3a and C3aR was observed in Factor B deficient mice in response to BSA (Figure S4A,B).

### *In vitro* studies

#### C3a induces PEC proliferation and migration

**Figure 5:**
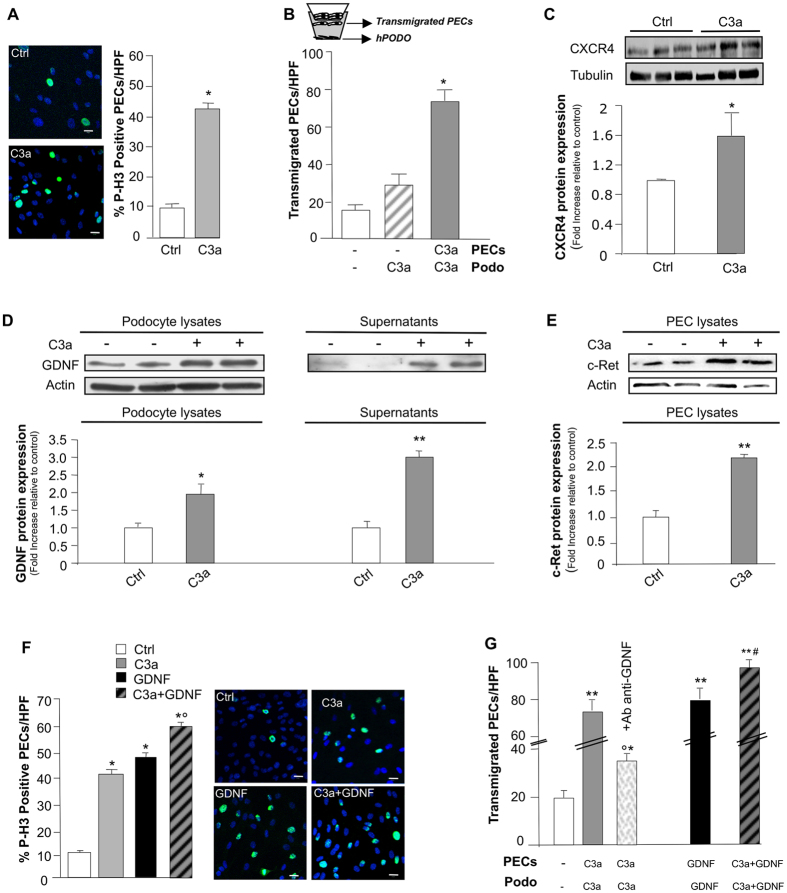
C3a-dependent PEC activation is enhanced by podocyte-derived GDNF *in vitro*. (**A**) Representative images of P-H3 (green) and DAPI (blue) in untreated (Ctrl) or C3a-treated PECs (24 hours). Quantification of proliferating PECs (P-H3^+^) exposed to medium or C3a (1 μM). Data expressed as percentage of P-H3^+^PECs per total DAPI-positive cells/HPF (5–8 random fields, *n* = 5 experiments). *P < 0.001 versus Ctrl. (**B**) Quantification of cell migration in co-culture transwell system of podocytes and PECs (see scheme) exposed to medium or C3a. Data expressed as number of PECs migrated from the upper chamber across the filter towards podocytes/HPF (5–8 random fields, *n* = 4 experiments). *P < 0.001 versus Ctrl and C3a-Podo. (**C)** Representative Western blotting and densitometric analysis of CXCR4 protein in untreated or C3a-stimulated PECs (15 hours). Tubulin used as sample loading control (*n* = 3 experiments). *P < 0.05 versus Ctrl. (D) Western blotting and densitometric analysis of GDNF in untreated or C3a-activated podocyte lysates (left panel) and supernatants (right panel) 15 hours. Actin used as sample loading control (*n* = 4 experiments). *P < 0.05, **P < 0.01 versus Ctrl. (**E**) Western blotting and densitometric analysis of c-Ret in untreated or C3a-stimulated PEC lysates (15 hours). (*n* = 3 experiments). **P < 0.01 versus Ctrl. (**F)** Representative images of P-H3 (green) and DAPI (blue) in untreated, C3a-, GDNF- or C3a + GDNF-treated PECs (24 hours). Scale bar: 20 μm. Quantification of proliferating P-H3^+^PECs exposed to medium, C3a, GDNF (100 ng/ml) and C3a + GDNF. Data expressed as percentage of P-H3^+^PECs per total DAPI-positive cells/HPF (5–8 random fields, *n* = 5 experiments). *P < 0.001 versus Ctrl; °P < 0.01 versus C3a- and GDNF-treated PECs. (**G**) Quantification of PEC migration in co-culture transwells from the upper chamber towards unstimulated or C3a-activated podocytes (24 hours) in the presence or absence of the anti-GDNF antibody. In additional samples, PECs and podocytes were exposed to GDNF or C3a + GDNF (24 hours). Data expressed as transmigrated PECs/HPF (5–8 random fields, *n* = 4 experiments). *P < 0.05, **P < 0.001 versus unstimulated cells; °P < 0.001 versus C3a, GDNF and C3a + GDNF-treated PECs; ^#^P < 0.01 versus C3a- and GDNF-treated PECs. Data information: Values are presented as mean ± SEM. Scale bars: 20 μm. (**A,B,F,G**) ANOVA corrected with Bonferroni coefficient or (**C,D,E**) unpaired Student’s *t*-test.

To explore the mechanisms through which activated complement induced PEC dysregulation, we studied in an *in vitro* setting the effects of C3a on PEC phenotype. We used human PECs, here characterized as progenitor cells for their positivity for CD133, CD24, and CD106 and for their ability to differentiate towards podocytes (see Methods, and Figure S5). PECs, similarly to podocytes^52^,^53^ constitutively expressed C3aR, as revealed by RT-qPCR (threshold cycle value: 30.2 ± 0.1). The direct effect of activated complement proteins on PECs was assessed by studying cell proliferation in response to C3a and evaluating the number of cell nuclei positive for histone H3 (P-H3), a marker of mitosis. A significant increase in the percentage of proliferating PECs after C3a incubation was observed compared to unstimulated cells (Fig. 5A). That the C3a challenge could induce PEC migration towards C3a-activated podocytes was demonstrated using a transmigration assay in a transwell system consisting of human PECs co-cultured with C3a-treated podocytes (Fig. 5B). The migration of C3a-stimulated PECs from the upper chamber across the membrane towards activated podocytes was significantly higher than that observed with untreated PECs (Fig. 5B). The migration of PECs induced by C3a was associated with an increased expression (P < 0.05) of CXCR4 (Fig. 5C).

#### C3a-dependent PEC dysregulation is potentiated by podocyte-derived GDNF

We next assessed if PEC dysregulation/activation also occurred via C3a-activated podocyte signals. We previously showed that C3a induced phenotypic changes in cultured human podocytes, in terms of migration, altered expression of F-actin–associated proteins, and Snail, a marker of dedifferentiation^54^. Here we focused on GDNF and its receptor c-Ret, both described as being upregulated in injured podocytes^55^. We found that GDNF was constitutively expressed by podocytes at low levels and was significantly increased in response to C3a in both cell extracts and supernatants (Fig. 5D). Unstimulated PECs also expressed c-Ret, which was significantly upregulated by C3a (Fig. 5E). When PECs were exposed to GDNF or C3a alone the percentage of proliferating cells increased significantly compared to unstimulated cells (Fig. 5F). The co-incubation of PECs with GDNF and C3a further enhanced their ability to proliferate (P < 0.01). The role of podocyte-derived GDNF in PEC migration rests on data from a co-culture system showing that incubation of both cell populations with C3a, in the presence of functional blockade of podocyte-derived GDNF with a specific antibody, significantly reduced PEC transmigration towards activated podocytes (Fig. 5G). The effect of GDNF *per se* on PEC motility was provided by data showing that GDNF increased PEC migration through the transwell filter (Fig. 5G). The combined exposure of PECs and podocytes to GDNF and C3a resulted in a more pronounced PEC migration compared to individual stimuli (P < 0.01) (Fig. 5G).

#### C3 deposits are associated with PEC activation and upregulation of CXCR4 and GDNF in glomeruli of patients with proteinuric nephropathy

**Figure 6:**
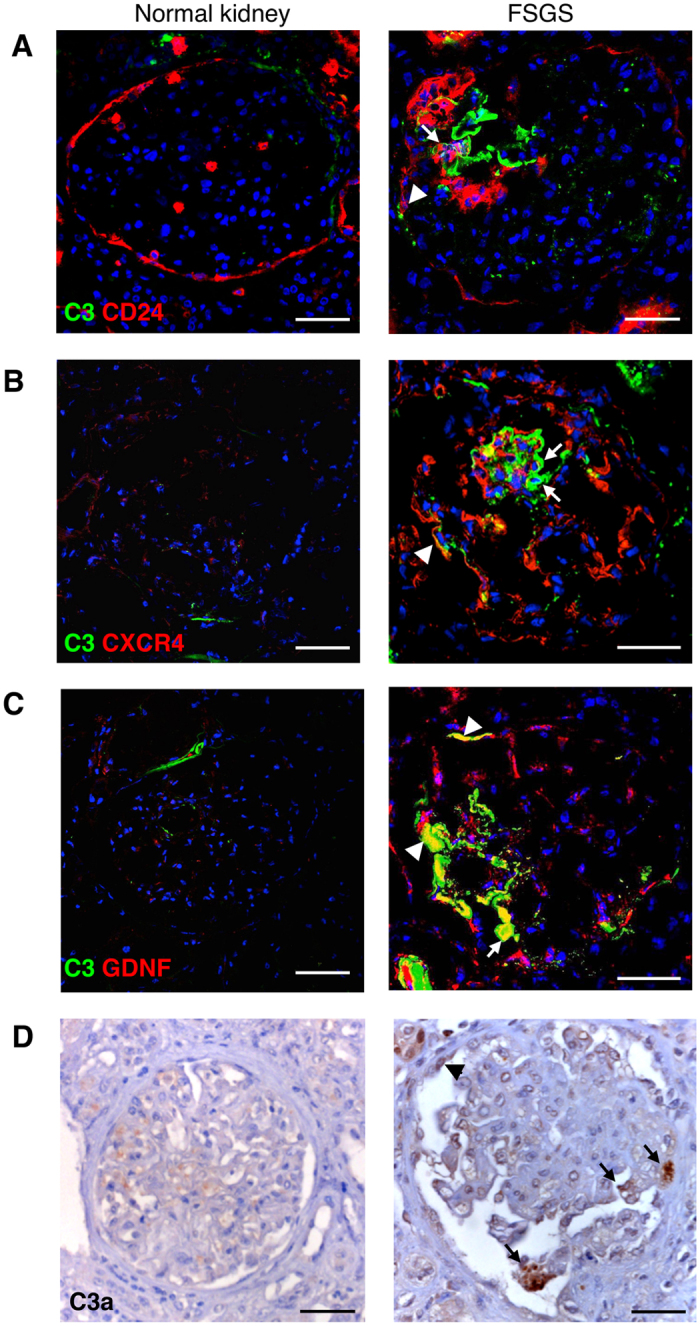
C3 and C3a, CD24, CXCR4 and GDNF expression in renal biopsies of patients with focal segmental glomerulosclerosis (FSGS). (**A**) Representative double immunofluorescence staining for C3 (green) and PECs labelled for CD24 (red) in normal control kidney (*n* = 4) and in the renal biopsy of patients with FSGS (*n* = 10). (**B,C**) Co-staining for CXCR4 (red) and C3 (green) (**B**), and GDNF (red) and C3 (green) (**C**) in normal subjects and FSGS patients. (**D**) Representative images of C3a immunostaining of renal tissue in normal and diseased kidneys. Data information: Podocytes and PECs are indicated with arrows and arrowheads, respectively. DAPI (blue) stains nuclei. Scale bars: 50 μm.

To examine the clinical relevance of the mechanisms described above, we took advantage of renal tissue from ten proteinuric patients with focal segmental glomerulosclerosis. Clinical characteristics are outlined in Table 1. We found that in proteinuric patients, glomerular C3 deposits were associated with the presence of exuberant accumulation of activated CD24-positive PECs (Fig. 6A, right and Figure S6) between the Bowman’s capsule and the glomerular capillary tuft. Similar to what observed in mice, abnormal C3 deposition was associated with increased staining of CXCR4 in the context of glomerular lesions (Fig. 6B, right and Figure S6) and overexpression of GDNF (Fig. 6C, right and Figure S6). Glomerular C3a staining was also observed (Fig. 6D). At variance, in normal renal tissue PECs were localized along the Bowman’s capsule in the presence of weak glomerular C3 and C3a staining (Fig. 6, left). No signal for CXCR4 and GDNF was detected in the normal glomeruli (Fig. 6B,C, left).

## Discussion

Clinical and experimental evidence documented that injury and loss of podocytes are critical determinants of PEC dysregulation in glomerular diseases/podocytopathies^19^,^27^,^28^,^29^,^31^. Having previously established that ultrafiltered complement plays a role in mediating podocyte damage (i.e. swelling, protein droplet accumulation and foot process fusion) in mice with protein-overload proteinuria^36^, here we show that abnormal glomerular C3 activation and accumulation exerted a potent toxic effect on podocytes resulting in their depletion in WT mice and even more in C*fh*^−/−^ mice. Importantly, the proof that impaired podocyte adhesion to the GBM in mice with protein-overload was functionally linked to the AP of complement activation derived from data that factor B deficiency prevented podocyte detachment. Moreover, no or trace glomerular C4 deposits in WT and C*fh*^*−/−*^ mice in response to BSA excluded the involvement of the classical pathway of complement activation in this model. In this context, there is evidence that complement via AP directly affects podocyte attachment to the GBM by altering integrin signals and cytoskeletal proteins in a murine model of podocyte injury induced by a bacterial toxin^54^.

The present results also detail a novel finding suggesting that complement activation at glomerular level contributes to activation of PECs. Exaggerated C3 deposition in WT mice, which was exacerbated in C*fh*^−/−^ mice, was associated with a high percentage of glomeruli developing synechiae and hyperplastic-like lesions. Podocytes and, to a major extent, PECs expressing claudin-1 and NCAM were present in the glomerular lesions. Data that blockade of factor B restored the distribution along the Bowman’s capsule of PECs with progenitor cell phenotype, clearly demonstrate the critical role of PECs with mature and immature phenotype in the sequence of events leading to sclerosis in response to complement AP activation. Importantly, in the glomeruli of both WT and C*fh*^−/−^ mice with protein-overload the presence of C3a -an active fragment generated when C3 convertase cleaves C3 in C3b-coupled to the increased expression of C3aR by podocytes and PECs may locally contribute to amplify cell injury. In this context, the relevance of C3a/C3aR axis has been documented as a part of a novel mechanism of renal fibrosis and glomerular sclerosis in animals and patients with diabetic nephropathy^56^,^57^.

To gain insight into the possibility that activated complement cascade represents a major mechanistic factor which influences podocyte and PEC behaviour and their interaction, different experimental settings have been employed. We show that cultured PECs proliferated and migrated towards activated podocytes in response to C3a. Moreover, C3a induced on PECs the upregulation of CXCR4, a promigratory factor described to be expressed by PECs forming hyperplastic lesions in proliferative glomerulonephritis^31^. These data, together with the *in vivo* evidence that glomerular Ki-67 and CXCR4 expression increased in WT and C*fh*^−/−^ mice and was normalized in B*f*^−/−^ mice with protein-overload, imply the involvement of complement activation via AP in the enhanced proliferation and CXCR4-dependent migration of dysfunctioned PECs, thereby contributing to glomerular hypercellular lesions.

The aberrant behaviour of PECs in response to complement was further amplified by factors released by complement-activated podocytes. When podocytes were exposed to C3a, they expressed and released GDNF, conceivably as an adaptive response. This observation relies on a study highlighting the role of GDNF and its receptor c-Ret, as critical factors for survival response of podocytes during toxic injury^55^. Here, the biological relevance of the GDNF/c-Ret pathway rests on data showing that C3a upregulated c-Ret on PECs and that in co-culture C3a-activated podocytes induced migration of PECs through the release of GDNF. Notably, exogenously added GDNF triggered the proliferation and migration of PECs and potentiated the C3a effects. This observation raises the more general possibility that in proteinuric conditions the toxic effects of ultrafiltered complement components on PECs could be further enhanced through the GDNF/c-Ret axis. As proof of this mechanism, in mice with protein-overload, the increased staining of GDNF was paralleled by upregulation of c-Ret in both podocytes and PECs. The present findings in mice had an important clinical correlate in ten patients with proteinuric nephropathy characterized by activated CD24-positive PECs, who exhibited glomerular accumulation of C3 and C3a associated with high expression of both CXCR4 and GDNF in the glomerular lesions.

Together our data underline the importance of the aberrant complement activation via AP at glomerular level as trigger of podocyte-dependent PEC dysregulation, and offer new insights into therapeutic strategies that aim to inhibit the complement system in proteinuric glomerular diseases. Among strategies aimed at intercepting C3, derivatives of the compstatin family and soluble CR1 displayed favorable effects in preclinical models and in patients with age-related macular degeneration or C3 glomerulopathy^58^,^59^,^60^. However, targeted C3 inhibition often raised the concern that a completely shut down of C3 activity could increase susceptibility to infections. Other complement regulator-based approaches blocking the activation of C3 via the AP, such as TT30 (CR2- FH fusion protein) and mini-FH^58^, as well as C3a receptor antagonists, tested in preclinical studies^56^,^57^ could offer the advantage of avoiding the adverse effects, providing unprecedent opportunities for long-term systemic interventions.

In summary, in protein-overload proteinuria we found that 1. Aberrant glomerular accumulation of C3 and C3a is not dispensable to podocyte dysfunction and loss leading to PEC activation; 2. Mice genetically deficient in factor H and B provided proof of concept that complement activation occurs via AP; 3. Proliferation and migration of PECs in response to activated complement and to podocyte-derived GDNF contribute to the development of glomerular sclerotic lesions. Mechanistic *in vitro* studies clearly established the involvement of C3a/C3aR in podocyte-dependent PEC dysfunction. Consistently, renal biopsies from patients with proteinuric nephropathy and PEC activation showed concomitant glomerular C3 and C3a deposition and high expression of CXCR4 and GDNF, indicating a key role of C3/C3a in the development of glomerular lesions. Given that novel therapies targeting complement have recently been approved for complement-related diseases it is worth considering that their use could be extended to patients with progressive proteinuric nephropathies.

## Methods

### Animal experiments

All procedures involving animals were performed in accordance with institutional guidelines in compliance with national (D.L.n.26, March 4, 2014), and international laws and policies (directive 2010/63/EU on the protection of animals used for scientific purposes) and were approved by the Institutional Animal Care and Use Committees of Mario Negri Institute. Animals were housed in a constant-temperature room with a 12-hour dark/12-hour light cycle in specific pathogen free facility, and fed a standard diet. Factor H-deficient (C*fh*^−/−^) and Factor B-deficient (B*f*^−/−^) mice^44^,^45^ from a C57BL/6 genetic background and WT littermates were kindly provided by Drs. Matthew C. Pickering and Marina Botto (Imperial College, London). Matings for obtaining heterozygous mice for factor H and factor B were performed at Mario Negri Institute. Genotypes were determined by PCR^44^,^45^. Protein-overload proteinuria was induced in five month old male C*fh*^−/−^, B*f*^−/−^ mice and corresponding WT littermates as previously described^36^, with slight modifications. Unilateral nephrectomy was performed 5 days before starting injections of bovine serum albumin (BSA, day 0). Mice received i.p. injections of 5 and 10 mg/g body weight of BSA (A-7906, Sigma-Aldrich) the first two days, then BSA was given at 15 mg/g body weight five days-weekly for three weeks. Mice were sacrificed on day 23 after starting BSA. Control C*fh*^−/−^, B*f*^−/−^ mice and corresponding WT littermates received the same volume of saline. Urinary albumin/creatinine ratio was calculated by measurement of albuminuria by ELISA test (Albuwell M test kit, Exocell) and creatininuria by a Cobas Mira autoanalyzer (Roche Diagnostics Systems). The BSA-overload model is characterized by proteinuria with no evidence of electron dense deposits within the kidney thus excluding renal immune complex deposition^36^,^42^.

### Renal histology

Duboscq-Brazil-fixed paraffin-embedded kidney sections (3 μm) were stained with periodic-acid Schiff (PAS) reagent. At least 80 glomeruli were examined for each animal, and the extent of lesions was expressed with a score from 0 to 4 related to the percentage of the Bowman’s capsule area occupied by the lesions (0: no lesions, 1: lesions affecting ≤25% of the glomerulus, 2: lesions affecting 25 to 50% of the glomerulus, 3: lesions affecting 51 to 75% of the glomerulus, and 4: lesions affecting 76 to 100% of the glomerulus). The extent of glomerular damage was expressed as the percentage of sclerotic glomeruli. All biopsies were reviewed by a blinded pathologist.

### Renal ultrastructural analysis

Fragments of kidney tissue were processed as described^54^. Ultrathin sections were stained with uranyl acetate and lead citrate and examined using a transmission electron microscope (Morgagni 268D, Philips, Brno, Czech Republic).

### Immunofluorescence analysis in renal tissue

OCT-frozen mouse and human renal sections, and periodate-lysine-paraformaldehyde (PLP)-fixed frozen mouse samples, after antigen retrieval, were incubated with primary antibodies (see Supplementary Methods) followed by FITC or Cy3-conjugated secondary antibodies (Jackson ImmunoResearch Laboratories, West Grove, PA). Nuclei were stained with DAPI and renal structure with lectins, either rhodamine lens culinaris agglutinin or FITC-wheat germ agglutinin (WGA) (Vector Laboratories, Burlingame, CA). Negative controls were obtained by omitting the primary antibody on adjacent sections. Samples were examined using an inverted confocal laser microscope (LSM 510 Meta; Zeiss, Jena, Germany) or with ApoTome Axio Imager Z2 (Zeiss). C3 deposits and nestin staining were quantified by using ImageJ software (National Institutes of Health, Bethesda, MD) expressing the positive glomerular areas as a percentage of the total area. At least 10–15 glomeruli/section for each animal were randomly analyzed.

### Cell cultures and characterization

#### Podocytes

Conditionally immortalized human podocytes (kindly provided by dr. P. Mathieson and dr. M.A. Saleem, Children’s Renal Unit and Academic Renal Unit, University of Bristol, Southmead Hospital, Bristol) were differentiated as described^61^.

#### PEC isolation

Human PECs were isolated, as described^62^, from a normal portion obtained from the tumor nephrectomy of a 3-year-old patient. The kidney fragment was digested with collagenase IV (750 U/ml; Sigma) for 45 minutes at 37 °C, the cell suspension was centrifuged and the pellet was cultured in endothelial cell growth medium-MicroVascular (EGM-MV) (Lonza Sales Ltd.) plus 20% fetal bovine serum (FBS) (Thermoscientific Hyclone).

#### PEC characterization

After 4 days of culture, CD133^+^ fraction was recovered by immunomagnetic separation of total renal cells using CD133 Cell Isolation Kit (Miltenyi)^62^. Clones were generated from CD133^+^ cells by limiting dilution in 96-well plates and immunofluorescence and FACs analysis were used to identify clones positive for CD24 (Santa Cruz Biotechnology) and CD106 (Sigma-Aldrich) (Figure S5A–C). One CD133^+^ CD24^+^ CD106^+^ clone was then characterized for the ability to differentiate toward podocytes, and used for all *in vitro* experiments. Podocyte differentiation was obtained by culturing cloned PECs for 6 and 24 hours with VRAD medium^18^. PECs exposed to the VRAD medium exhibited a significant increase in the podocyte markers *CD2AP* and *NEPHRIN*, as determined by RT-qPCR (Figure S5D). Immunofluorescence analysis confirmed that PECs exposed to VRAD medium were positive for α-actinin-4, CD2AP and nephrin (Figure S5E).

### Immunofluorescence studies *in vitro*

PECs were fixed in 2% paraformaldehyde, permeabilized with Triton 0.3% and incubated with primary (see Supplementary Information) followed by secondary antibodies (1:100; Jackson ImmunoResearch Laboratories). Nuclei were counterstained with DAPI. Proliferation was evaluated by studying the phosphorylation at Ser10 of histone H3 in PECs ref. 63 incubated with medium alone or in the presence of C3a (1 μM, Millipore) or GDNF (100 ng/ml, Abcam) for 24 hours. The number of P-H3-positive cells was counted in randomly acquired 5–8 fields/sample (magnification X400), normalized for the number of DAPI positive cells and expressed as the percentage of P-H3-positive PECs per total PECs in each HPF.

### Patients

Renal tissues of ten patients with focal segmental glomerulosclerosis (FSGS) from the archives of the Unit of Nephrology, Azienda Ospedaliera Papa Giovanni XXIII (Bergamo, Italy) were studied. Demographic and clinical parameters (proteinuria and serum creatinine levels, and glomerular filtration rate estimated by the “Chronic Kidney Disease Epidemiology Collaboration” equation, CKD-Epi formula^64^) at the time of renal biopsy were retrieved from the hospital database. Specimens of uninvolved portions of kidney collected from tumor nephrectomy (n = 4 patients) were used as controls. All experimental protocols involving human subjects and requiring informed consent are carried out in accordance with the Declaration of Helsinki and good clinical practise guidelines, and approved by the Ethical Committee of the Clinical Research Center of the IRCCS-Istituto di Ricerche Farmacologiche Mario Negri and the Azienda Socio Sanitaria Territoriale (ASST) Papa Giovanni XXIII.

### Statistical analysis

Results are expressed as means  ±  SEM. Data analysis was performed with Prism Software (GraphPad Software Inc.). Comparisons were made using ANOVA with the Tukey or Bonferroni post hoc test, or unpaired Student’s *t*-test, or non-parametric Mann-Whitney test, as appropriate. Statistical significance was defined as P < 0.05.

## Additional Information

**How to cite this article**: Morigi, M. *et al*. A previously unrecognized role of C3a in proteinuric progressive nephropathy. *Sci. Rep.* **6**, 28445; doi: 10.1038/srep28445 (2016).

## Supplementary Material

Supplementary Information

## Figures and Tables

**Table 1:** Demographic, clinical and histopathological characteristics of patient populations.

**Patients,** ***n***	**10**
Age, *years-range*	24–58
Sex, *Male:Female*	7:3
SBP, *mmHg*	122.1 ± 10.4
DBP, *mmHg*	77.5 ± 6.5
Proteinuria, *g/day*	10.5 ± 9.6
Serum creatinine, *mg/dL*	1.6 ± 1.1
GFR, *ml/min/1.73m*^2^	72.7 ± 37.4

SBP: systolic blood pressure; DBP: diastolic blood pressure; GFR: glomerular filtration rate.

Data are expressed as mean ± SD.
