# Supplementary material for: A previously unrecognized role of C3a in proteinuric progressive nephropathy
Source: Sci Rep. 2016 Jun 27;6:28445. doi: 10.1038/srep28445 (PMC4921969; doi:10.1038/srep28445)
Supplement: Supplementary Information [file srep28445-s1.pdf]

SUPPLEMENTARY INFORMATION

A previously unrecognized role of C3a in proteinuric progressive nephropathy

Marina Morigi[1], Monica Locatelli[1], Cinzia Rota[1], Simona Buelli[1], Daniela Corna[1], Paola Rizzo[1], Mauro Abbate[1], Debora Conti[1], Luca Perico[1], Lorena Longaretti[1], Ariela Benigni[1], Carlamaria Zoja[1], Giuseppe Remuzzi[1-3]

[1]IRCCS - Istituto di Ricerche Farmacologiche “Mario Negri”, Centro Anna Maria Astori, Science and Technology Park Kilometro Rosso, Bergamo, Italy

[2]Unit of Nephrology and Dialysis, Azienda Ospedaliera Papa Giovanni XXIII, Bergamo, Italy

[3]Department of Biomedical and Clinical Sciences, University of Milan, Milan, Italy

Supplementary Methods

List of primary antibodies for immunofluorescence analysis

OCT-frozen mouse kidney sections:

- goat FITC-conjugated anti-mouse C3 (1:200; Cappel Durham, NC)
- goat anti-nephrin (1:100; Santa Cruz Biotechnology Inc., Santa Cruz, CA)
- rat anti-nestin (1:300; Abcam, Cambridge, UK)
- rabbit anti-claudin-1 (undiluted; Thermo Scientific, Rockford, IL)

PLP-fixed frozen mouse kidney sections:

- rabbit anti-NCAM (1:1000; Millipore, Billerica, MA)
- rabbit anti-Ki-67 (1:200; Abcam)
- rabbit anti-CXCR4 (1:50; Abcam)
- rabbit anti-GDNF (1:100; Santa Cruz Biotechnology Inc.)

OCT-frozen human kidney sections:

- rabbit FITC-conjugated anti-human C3 (1:25; Dako, Glostrup, Denmark)
- mouse anti-CD24 (1:25; Santa Cruz, Biotechnology Inc.)
- rabbit anti-CXCR4 (1:50; Abcam)
- rabbit anti-GDNF (1:200; Abcam) antibodies

Immunofluorescence studies *in vitro*

- rabbit anti α-actinin-4 (1:200; Origene)
- rabbit anti-CD2AP (1:300; Santa Cruz Biotechnology)
- goat anti-nephrin (1:100; Santa Cruz Biotechnology)
- rabbit anti-phospho (P) histone H3 (1:75; Cell Signaling)

Immunoperoxidase analysis in renal tissue

Duboscq-Brazil-fixed, 3μm paraffin embedded kidney sections were incubated with goat anti-c-Ret (1:100; Santa Cruz Biotechnology Inc.) and mouse anti-C3a/C3a des Arg (1:100; Abcam) antibodies followed by corresponding HRP-Polymer kit (Biocare Medical, Concord, CA) and DAB (Biocare Medical) substrate solution. C3a receptor was detected by chicken anti-mouse C3aR antibody (1:200; BMA Biomedicals, Augst, Switzerland) followed by HRP-conjugated goat anti-chicken antibody (1:400; Jackson Immunoresearch Laboratories) using AEC immunoperoxidase technique. **To detect C4, a goat anti-C4α (1:100; Santa Cruz Biotechnology) was used, followed by biotinylated horse anti-goat secondary antibody (1:200, Jaskson Immunoresearch), alkaline phosphatase-conjugated streptavidin, and Warp Red (Biocare Medical) substrate solution.** Slides were finally counterstained with hematoxylin and observed by light microscopy (ApoTome, Axio Imager Z2, Zeiss). Negative controls were obtained by omitting the primary antibody on adjacent sections.

Glomerular podocyte quantification

Podocytes were identified as cells positive for Wilms' tumor 1 (WT1). OCT-frozen kidney sections were incubated with rabbit anti-WT1 antibody (1:400; Santa Cruz Biotechnology), followed by Cy3-conjugated goat anti-rabbit IgG (1:50; Jackson Immunoresearch Laboratories). Nuclei and cell membranes were stained with DAPI and FITC-WGA lectin. At least 30 glomeruli/section for each animal were randomly acquired using confocal laser scanning microscope. The estimation of the average number of podocytes per glomerulus was determined by morphometric analysis as previously described (Macconi D et al., Am J Pathol 2006, 168: 42-54). In addition, quantification of podocyte area was performed by evaluating nestin expression with a rat anti-mouse nestin (1:300, Abcam) followed by FITC goat anti-rat (1:100, Jackson Immunoresearch Laboratories) antibody. Data are expressed as % of nestin positive staining/glomerular area.

RT- qPCR

Human *CD2AP* and *NEPHRIN* mRNA expression in cloned PECs were determined by real-time PCR. Total RNA was isolated as previously described {Morigi, 2006 #348}. To amplify human cDNAs, we used SYBR Green PCR Master Mix (Applied Biosystems) and the following primers: *CD2AP* (300nM) forward 5'- CCCTGGATGAACTTAGAGCCC -3' reverse 5'- TCCAGTTCTTTCCCGTGATCC -3'; *NEPHRIN* (300nM) forward 5'- GGCCACAGCCAGGGTGA -3' reverse 5'- ATGGGGGCCTCCAGTGC -3'; *HPRT* (Hypoxanthine phosphoribosyltransferase, housekeeping gene, 300nM) forward 5'- GGCAGTATAATCCAAAGATGGTCA-3' reverse 5'-TCCTTTTCACCAGCAAGCTTG-3'. The PCR was performed on the Viia7 Real Time PCR System (Applied Biosystems). We used the ΔΔCt technique to calculate cDNA content in each sample using the cDNA expression in PECs without VRAD as calibrator.

Western blot analysis

Samples were processed as previously described (Locatelli M et al., J Am Soc Nephrol 2014, 25, 1786-98). Supernatants were collected and centrifuged (1200 rpm, 10 minutes) to remove cell debris. Proteins were then concentrated by cold acetone (Sigma-Aldrich). After drying, the protein pellets were dissolved as previously described (Locatelli M et al., J Am Soc Nephrol 2014, 25, 1786-98). Membranes were incubated with rabbit polyclonal antibodies against GDNF (1:200; Santa Cruz Biotechnology), c-Ret (1:200; Santa Cruz Biotechnology), CXCR4 (1:200; Abcam) or against actin (1:6000; Sigma-Aldrich) or tubulin (1:1000; Sigma-Aldrich) overnight at 4°C followed by a specific IgG-HRP secondary antibody (1:30000; Sigma-Aldrich). Bands were visualized as previously described (Locatelli M et al., J Am Soc Nephrol 2014, 25, 1786-98), and actin or tubulin were used as sample loading control. The GDNF bands in supernatants were normalized to the number of cells in each sample.

Migration assay

PECs were seeded in a transwell chamber on the upper side of an 8-μm permeable membrane, whereas podocytes were co-cultured in the lower chamber. Podocytes and/or PECs were incubated for 24 hours with medium alone or in the presence of C3a (1 μM), GDNF (100 ng/ml) alone or in combination with C3a. Additional samples incubated with C3a were exposed to GDNF-neutralising antibody (5 mg/mL; Abcam). PECs that migrated to the lower side of the filter were fixed in 11% glutaraldehyde and stained with hematoxylin and eosin. The number of migrated cells in 5-8 random fields (magnification X10) for each filter was counted. Data were expressed as number of migrated PECs/HPF.

Supplementary Figure Legends

Figure S1.

A. Representative images (left panel) and quantification of nestin expression (right panel) in wild type C*fh*$^{+/+}$ or B*f* $^{+/+}$ injected with saline or BSA. Scale bars: 20 μm. Values are mean±SEM (*n*=4 animals/group), *P<0.05 versus corresponding saline, Mann-Whitney test.

B. Representative transmission electron micrographs of kidney sections from C*fh*$^{-/-}$ mice injected with saline (left) or BSA (right) on day 23. Subendothelial electron dense deposits were seen (black arrows) in both groups. Protein droplets (arrowheads), vacuoles (asterisks), prominent cytoskeleton rearrangement, effacement of foot processes (white arrows), and microvillous transformation were present in podocytes in C*fh*$^{-/-}$ mice injected with BSA. Scale bars: 2000 nm.

Figure S2.

Representative images of kidney sections stained for glomerular C4 (red) in WT, C*fh*$^{-/-}$ or B*f* $^{-/-}$ mice injected with saline or BSA. Positive control: kidney section of mouse with chronic rejection. Scale bars: 20 μm.

Figure S3.

A-B. Representative images showing claudin-1 (A, green) and NCAM (B, red) expression in WT littermates of B*f*$^{-/-}$ mice injected with saline or BSA. Renal structure is stained with rhodamine (A, red) or FITC-WGA lectin (B, green).

C. Double immunofluorescence staining of claudin-1^{+} PECs (green) and nestin^{+} podocytes (red) in B*f* $^{-/-}$ mice injected with saline or BSA.

D. Representative images of Ki-67-positive cells (red) in B*f* $^{-/-}$ mice injected with saline or BSA. Renal structure is stained with FITC-WGA lectin (green).

E-F. Expression of CXCR4 (E) and GDNF (F) in kidney samples of WT littermates of B*f*$^{-/-}$ mice injected with saline or BSA. Renal structure is counterstained with FITC-WGA lectin (green). Nuclei are counterstained with DAPI **(A-F).** Scale bars: 20 μm **(A-F).**

G. Glomerular expression of c-Ret in podocytes (arrow) and PECs (arrowheads) by immunohistochemistry in WT littermates of B*f*$^{-/-}$ injected with saline or BSA Scale bars: 20 μm.

Figure S4.

A-B. Representative images of C3a (A) and C3a receptor (B) as detected by immunohistochemistry in C*fh*$^{-/-}$ and B*f* $^{-/-}$ mice injected with saline or BSA. Podocytes and PECs are indicated with arrows and arrowheads, respectively. Scale bars: 20 μm

Figure S5

A-C. Characterization of PECs: FACS analysis (A) for the expression of CD106 and CD24 of PECs after clonal expansion (right panel) obtained by limiting dilution of CD133+ fraction of renal cells recovered by immunomagnetic separation. Negative control is shown (left panel). Representative fluorescence histograms (B) showing the expression of CD106 and CD24 in cloned PECs. The negative control is shown (violet) (*n*=3 experiments). Representative images (C) of cloned PECs co-expressing CD106 (red) and CD24 (green) by confocal microscopy (merged image, yellow). Nuclei are counterstained with DAPI. Scale bars: 50 μm.

D. Differentiative potential of cloned PECs towards podocyte lineage evaluated by the expression of *CD2AP* and *NEPHRIN* mRNA using RT-qPCR. Cloned PECs were exposed for different time intervals (0, 6, 24 hours) to DMEM F12 supplemented with Vitamin D3 and retinoic acid (VRAD) medium. Values are mean±SEM (*n*=3 experiments). *P<0.01 versus 0 hours, ANOVA corrected with Bonferroni coefficient.

E. Representative images of the podocyte markers α-actinin-4 (α-ACT4), CD2AP and nephrin in cloned PECs after 24 hour exposure to VRAD medium (confocal microscopy). Nuclei are counterstained with DAPI. Scale bars: 20 μm.

Figure S6. Glomerular CD24, CXCR4 and GDNF expression in patients with FSGS. Representative images showing CD24, CXCR4 and GDNF expression in glomeruli of patients with FSGS. DAPI (blue) stains nuclei. Scale bars: 50 μm.

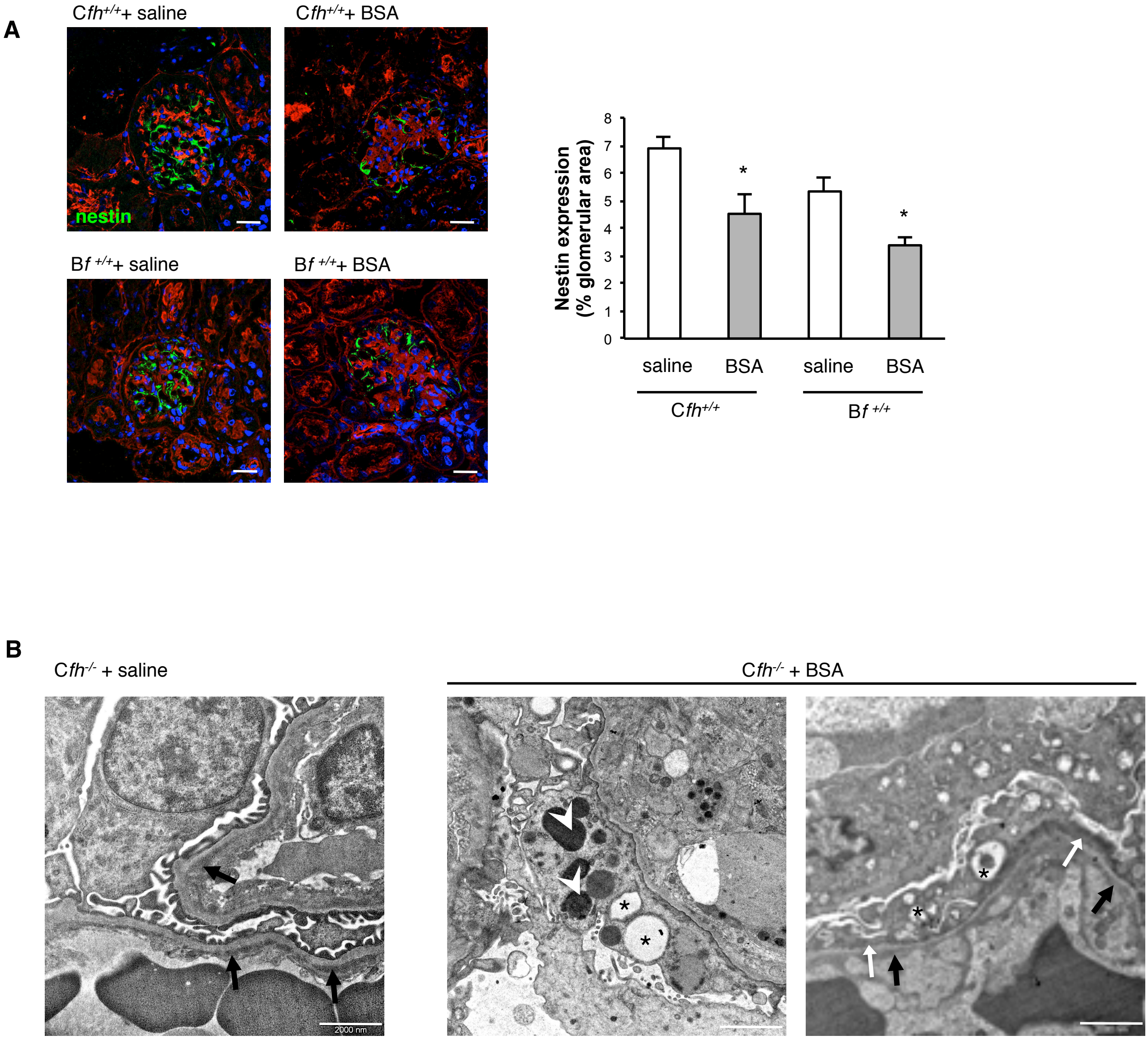
A
Cfh+/++ saline
Cfh+/++ BSA
nestin
Bf +/++ saline
Bf +/++ BSA
Nestin expression
(% glomerular area)
0
1
2
3
4
5
6
7
8
*
*
saline
BSA
saline
BSA
Cfh+/+
Bf +/+
B
Cfh-/- + saline
Cfh-/- + BSA
2000 nm

Figure S1

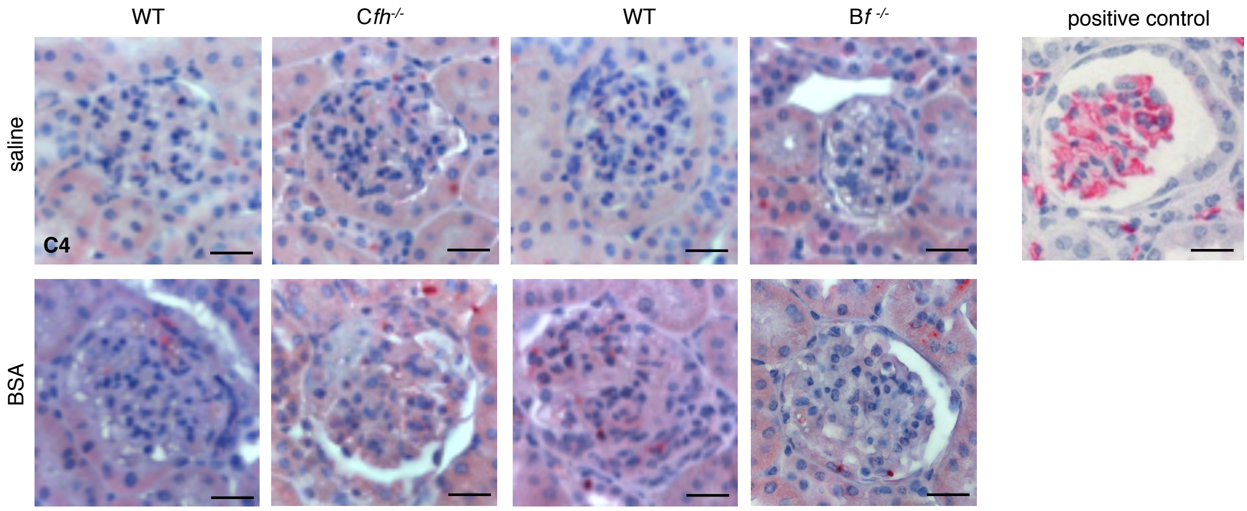

Figure S2

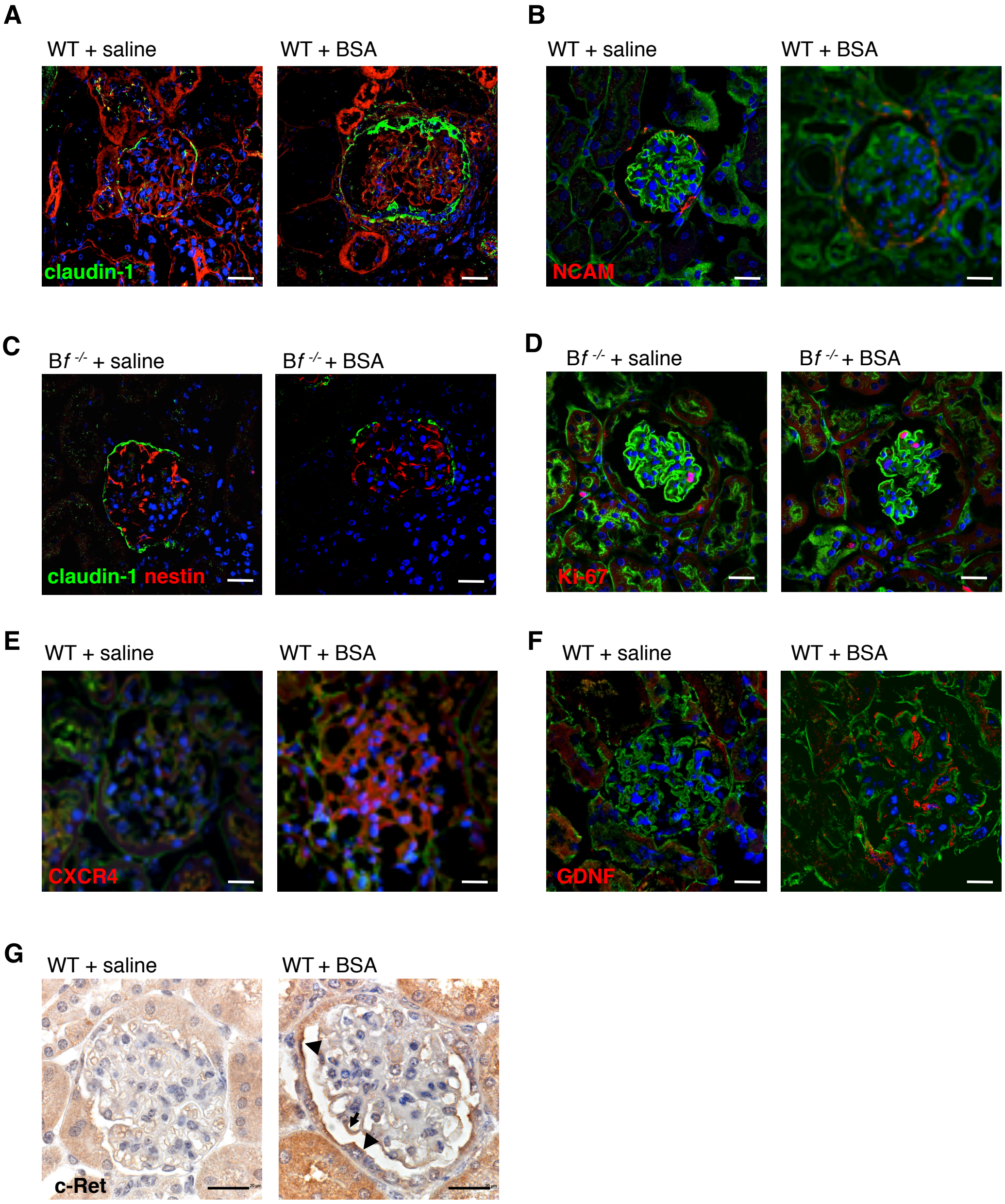
A
WT + saline
WT + BSA
claudin-1
B
WT + saline
WT + BSA
NCAM
C
Bf -/- + saline
Bf -/- + BSA
claudin-1 nestin
D
Bf -/- + saline
Bf -/- + BSA
Ki-67
E
WT + saline
WT + BSA
CXCR4
F
WT + saline
WT + BSA
GDNF
G
WT + saline
WT + BSA
c-Ret

Figure S3

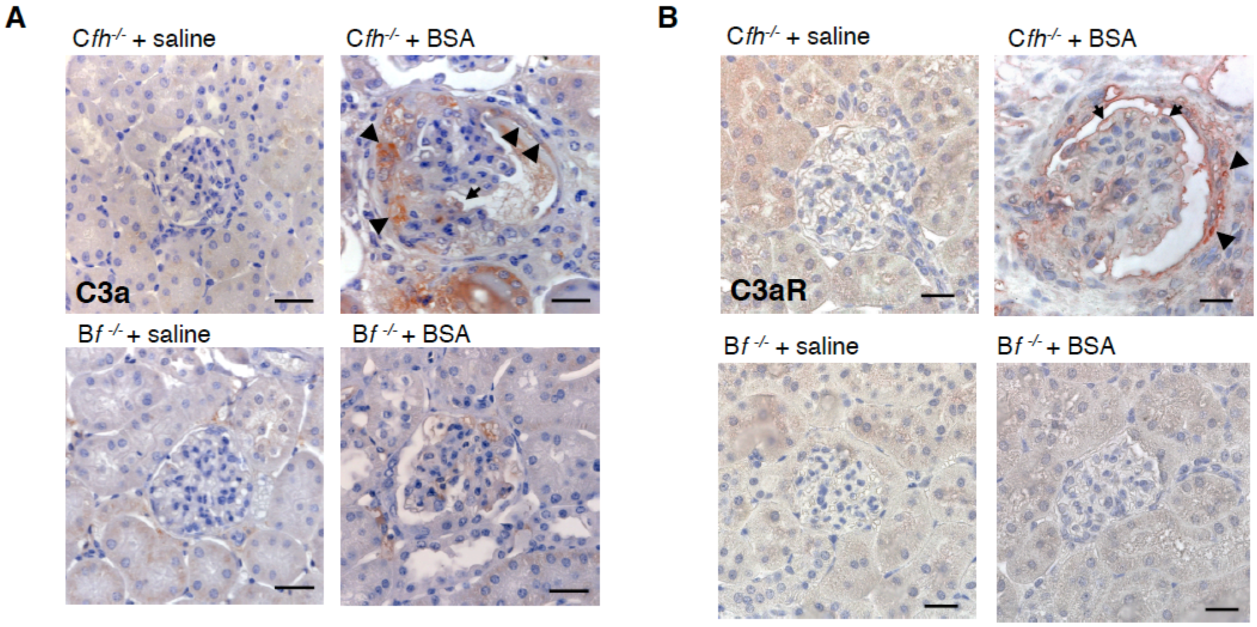
A
Cfh-/- + saline
Cfh-/- + BSA
C3a
Bf -/- + saline
Bf -/- + BSA
B
Cfh-/- + saline
Cfh-/- + BSA
C3aR
Bf -/- + saline
Bf -/- + BSA

Figure S4

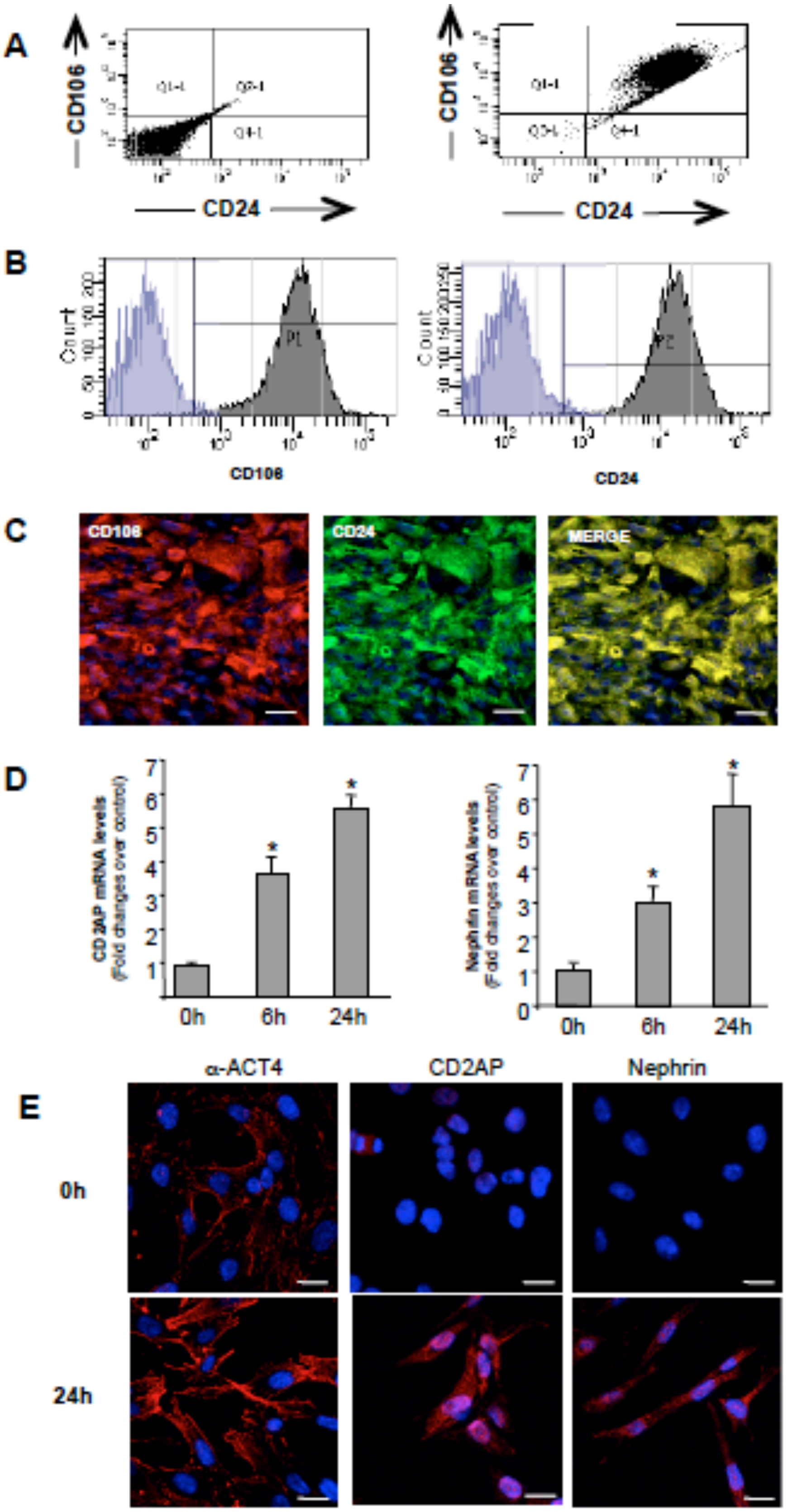
A
CD106
CD24
B
Count
CD106
CD24
C
CD106
CD24
MERGE
D
CD2AP mRNA levels (Fold changes over control)
Nephrin mRNA levels (Fold changes over control)
0h
6h
24h
E
α-ACT4
CD2AP
Nephrin
0h
24h

Figure S5

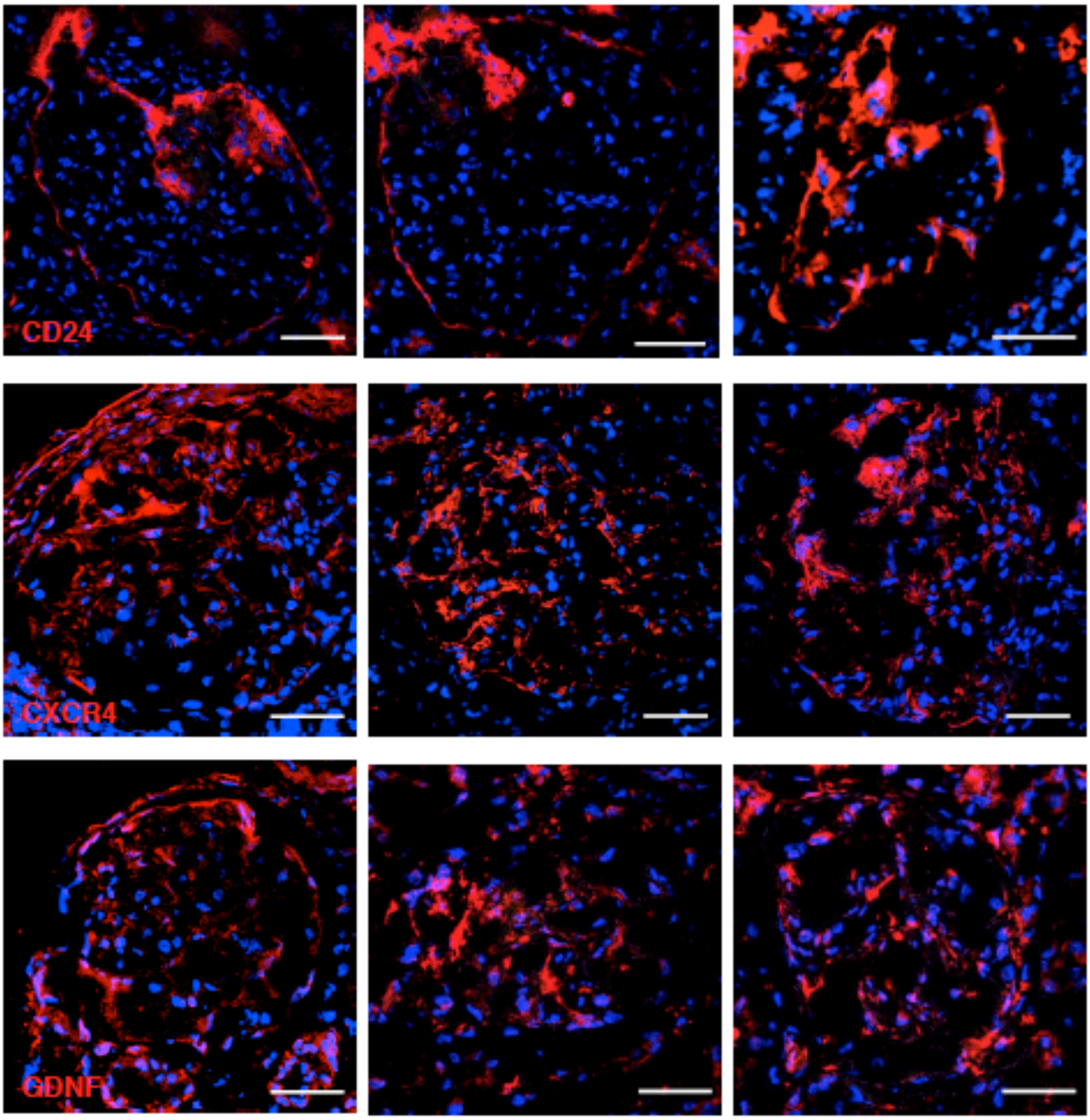

Figure S6
